# An Investigation into Coolant-Related Internal Diesel Injector Deposits from Heavy-Duty Vehicles

**DOI:** 10.1021/acsomega.4c11346

**Published:** 2025-06-04

**Authors:** Sarah L. Hruby, Pavlos Chrysafis, Henrik Kusar, Mayte Pach, Henrik Hittig

**Affiliations:** a Department of Chemical Engineering, 7655 KTH Royal Institute of Technology, Brinellvägen 8, Stockholm 114 28, Sweden; b Scania Technical Centre, Scania CV AB, Granparksvägen 10, Södertälje 151 48, Sweden

## Abstract

The formation of internal diesel injector deposits (IDIDs) in heavy-duty engines is a growing problem as engine technology becomes more advanced while fuel blends become more diverse, posing new challenges for mixing and compatibility. IDIDs have a variety of causes that can be challenging to pinpoint due to the number of factors involved, such as engine operation effects, fuel types, fuel additives, and fuel contamination. The aims of this study were to characterize IDIDs formed in an injector from an engine operating on a biofuel blend contaminated with coolant, gain a deeper understanding of the underlying formation mechanisms, and identify potential markers of coolant contamination in failed field injectors. In this study, a failed injector from the field was examined that was known to have fuel contamination from coolant. Laboratory experiments using the thermal deposit test (TDT) were carried out to generate deposits from a test fuel spiked with coolant. The laboratory and field deposits were characterized and compared using scanning electron microscopy with energy-dispersive X-ray spectroscopy (SEM-EDX), Fourier transform infrared attenuated reflectance spectroscopy (FTIR-ATR), and pyrolysis combined with gas chromatography (Py GC-MS). The results indicate that the deposits generated in the TDT were found to be primarily composed of sodium carboxylates originating from the organic acid technology additives in the coolant. The deposits were found to have structures with similarities to grease soaps, oleogels, or paraffin wax, suggesting that similar formation mechanisms may be involved. In contrast, the field injector deposits consisted of three distinct types: a cracked layer composed of sulfate salts and metal carboxylates, a globular cluster layer consisting of metal carboxylates, and particulate deposits that differ from the surroundings. The high proportion of sodium carboxylates in the globular cluster deposits was the key similarity to the laboratory deposits. In addition to the high sodium content, particulate deposits containing silicon and aluminum or aluminum and nitrogen were identified as potential markers of coolant contamination in IDIDs.

## Graphical abstract

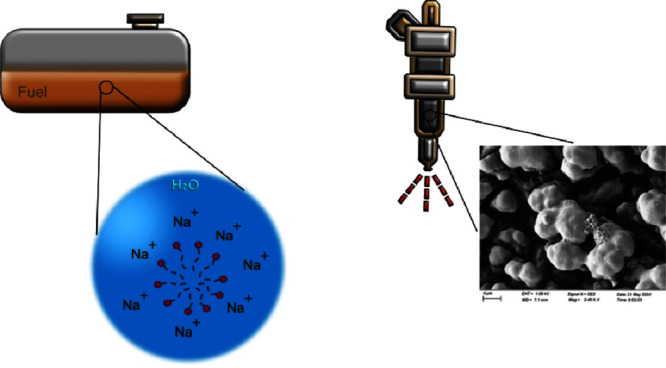

## Introduction

The formation of internal diesel injector deposits (IDIDs) can severely impact engine performance and lead to increased fuel consumption. Unlike external injector deposits, which can narrow or plug the nozzle holes, IDIDs interfere with the movement of the injection needle and disrupt both the amount and timing of fuel injection. The causes of IDIDs vary, but they generally include oxidized fuel or poor-quality fuel having poor stability and high acidity. Contamination of diesel fuel by salts, metals, and organic components from various sources have been shown to trigger IDID deposit formation.

Diesel fuels include conventional (fossil) diesel, biodiesel based on fatty acid methyl esters (FAME), renewable diesel, synthetic fuels (e.g., gas-to-liquid (GTL)), and various blends of these types. The diesel fuel mix continues to evolve as the adoption of biobased and renewable diesel increases globally. The total global production of biobased diesel is projected to increase from 952,000 barrels per day in 2021 to 1,382,000 barrels per day in 2028.^1^ Renewable diesel, also known as hydrogenated vegetable oil (HVO), comprised 22% of global biobased diesel consumption in 2022,^2^ and the demand for HVO continues to rise. Usage of FAME is often limited to blending into conventional diesel, with 7 vol % FAME (B7) being the most common blend in Europe. HVO is also used as a blending component in Europe without the 7 vol % limit that applies to FAME. FAME can be blended into diesel in higher proportions in certain countries, up to B30 (30 vol %).

Diesel standards vary depending on the market and the emission control requirements, becoming more stringent over time.^3^ While the most significant change in diesel has been the reduction of the sulfur content, other properties that impact the solvent power of the fuel, like boiling point range and total aromatics, have also been altered. Fuels with lower solvent power have a reduced capacity to dissolve impurities, such as water and metals, resulting in a greater tendency for deposit formation. Since different markets have different feedstocks and different standards, a vehicle may use different types of diesel or diesel blends when traveling between markets. This variation complicates the fuel mix within the tank and, subsequently, in the engine.

Contamination of fuel is a widespread problem, with many possible contaminants and entry points. Contamination may occur in fossil diesel or the FAME prior to blending, after blending prior to sale, during storage (such as at a depot), in the vehicle’s fuel tank, or within the vehicle’s fuel system. The most common contaminants seen in diesel fuel are water, engine oil, particulates, metals, and remnants from biodiesel production (e.g., glucosides, monoglycerides or soaps),^4–6^ but many others, such as gasoline, kerosene or coolant, are also possible.^7–10^

Water is a common contaminant in diesel fuel, entering through various pathways, such as tank contamination or moisture from the air.^5,11^ It can also result from fuel oxidation during aging.^12^ According to EN 590, the limit for water in diesel is 200 ppm (ASTM standard D6751). Biofuels are more hydrophilic than conventional fossil diesel (B0) and can contain up to 15 times more soluble water than B0, with levels from 1500 to 1980 mg/kg for the temperature range of 10 to 50 °C.^13^

Engine oil is a common diesel contaminant, particularly in engines with oil-lubricated high-pressure pumps.^14–16^ The main markers of oil contamination in fuel are calcium (from overbased detergents), phosphorus and zinc (from zinc dialkyl dithiophosphates), and sulfur (sulfuric acid formed in the engine).^17,18^

Coolant contamination of engine oil is a known problem.^19–21^ where ethylene glycol is reported to form lacquer inside engines via oxidation, esterification, and polymerization due to thermal degradation.^19,20^ Coolant contamination of fuel, however, is uncommon and has not previously been a focus of injector deposit studies. Coolant contamination can potentially occur in the fuel prior to fueling the vehicle or within the vehicle’s fuel system. Contamination within the vehicle depends on the heavy-duty fuel system and coolant system design, wherein for some systems, it is not possible to identify a plausible pathway. Consequently, contamination of the fuel prior to fueling or by human error are seen as more likely pathways. Ideally, the fuel filter will remove not only particulate matter from fuel, but also aqueous contaminants, such as coolant. However, not all fuel filters or systems have this capability. Additionally, if aqueous droplets are dispersed in the fuel, they may pass through the filter if sufficiently small (<10 μm).^22^ Organic acid technology (OAT) and hybrid organic acid technology (HOAT) are the dominant coolant types in use for diesel engines today. The organic acids, which may be aromatic, aliphatic monoacids, aliphatic diacids, or a combination of aromatic and aliphatic acids, provide corrosion protection in the radiator and throughout the coolant system. Coolants are typically composed of a 50/50 mixture of water and concentrate, where the concentrate contains the OAT or HOAT and glycol, usually ethylene glycol. Silicon-based HOAT contains metasilicate (SiO_3_^2–^) as a corrosion inhibitor, typically as sodium metasilicate. Other common components of coolant include: a bittering agent (denatonium benzoate), corrosion inhibitors (triazoles), and pH buffers. Other components may be included to improve the solubility of the additives in the glycol/water mixture. Coolants used in Asia contain phosphates but not silicates, while coolants used in Europe may contain silicates but lack phosphates. Combinations of silicates, phosphates, and OAT have been reported to be incompatible, leading to increased corrosion rates.^23^

Fuel contamination can lead to the formation of IDIDs, which can be classified based upon the nature and main constituents of the deposits. The list of deposit types below is based on that described by Edney et al.^24^

1. Metal carboxylates: These deposits, also known as soaps, are typically light in color, ranging from white to yellow and can be soft and then become hard and brittle when dry. The most common cations are sodium^25^ and calcium.^26^ Metal carboxylates form from acids in the fuel, originating via oxidation,^25,27,28^ hydrolysis,^5^ low-quality FAME containing free fatty acids,^29^ or additives.^30,31^ These fuel contaminants have been widely studied because they also clog fuel filters.^32,33^ Poor quality fuel is more susceptible to the formation of soap deposits, wherein as little as 0.1 mg/kg sodium has been noted as increasing deposits.^32^ Metal carboxylates can deposit in different ways, depending on the nature of the salt. Large aggregates of metal carboxylates, also known as soft particles, may deposit as particulates, while metal carboxylates with better solubility in diesel, such as sodium oleate, form more stable inverse micelles that collapse and precipitate on injector surfaces.^29,34^ An example of a metal carboxylate that has been shown to plug fuel filters and has therefore been used as a representative soft particle component is calcium methyl azelate (nonanedioate, C_9_).^35^ Other acids found to form metal carboxylates in fuel systems are octanedioic acid (suberic acid, C_8_), hexadecanoic acid (palmitic acid, C_16_), octadecanoic acid (stearic acid, C_18_), decanedioic acid (sebacic acid, C_18_), and icosanoic acid (arachidic acid, C_20_).^36^
2. Oxidized fuel: These sticky deposits result from low-temperature oxidation (autoxidation) of the fuel during aging, which includes the formation of acids, aldehydes, ketones, and alcohols – polar compounds with limited solubility in the fuel. Aging takes place both at low temperatures prior to use and within the vehicle. Autoxidation also occurs at elevated temperatures within the injector, as a small portion of the pressurized fuel is not injected and is returned to the fuel tank.^37^ During autoxidation, FAME can release free fatty acids via hydrolysis, form dimers via epoxidation and oligomerization, and can decompose into smaller molecules.^28,38–41^ As autoxidation of both renewable and fossil compounds proceeds, the soluble oxidation products grow into polymers that are no longer soluble in the fuel.^42,43^ The formation of deposits from autoxidation is the most common type of chemical reaction fouling.^43,44^

3. Polymeric: Polymeric deposits adhere strongly to injector sleeves and needles and can be classified based upon the key component. These deposits often contain oxidized fuel along with other elements, such as nitrogen or metals. Amide deposits are brown in color and result from the reaction of the fuel or engine oil additive, polyisobutylene succinimide (PIBSI) with fatty acids in the fuel. PIBSI-related deposits have been documented in relation to substandard fuel additive packages, where the PIBSI was of insufficient quality, or the additive package was not suited for the fuel’s solvent power.^15,45,46^ PIBSI deposits can also form when the fuel is contaminated with sodium and the succinimide ring opens at temperatures of 130–150 °C, forming a sodium carboxylate salt.^29^ Lacquer from fuel aging and oxidation are deposits that differ from oxidized fuel deposits in that the lacquer deposits are comprised of gums or resins that have reacted further with metal ions, such as sodium, resulting in a hard layer that tends to be thinner than other deposits such as metal carboxylates.^32^
4. Inorganic salts: Crystalline deposits with varying morphologies have been documented in IDIDs from field injectors. Sodium chloride deposits have been reported by various authors,^26^ including Ullmann et al.,^25^ who included an optical microscope image of the cube-shaped crystals on an injector needle and nozzle body. Calcium sulfate deposits were observed previously by Pach et al.^15,16,18^ Sodium carbonate deposits were reported by Trobaugh et al.^26^ and sodium sulfate deposits have been reported by Barker et al.^47,48^

5. Carbonaceous: These deposits are fine particulates and black in color, resulting from soot or coking.^49–52^ Carbonaceous deposits inside injectors are not typically associated with degraded engine performance and injector failure but are a known problem for external injector deposits.^29^

In this study, a failed injector from the field known to have had fuel contamination by coolant has been analyzed, and laboratory experiments were carried out in order to understand what kind of deposits can form from coolant-contaminated fuel. The aim of this study was not to exactly reproduce deposits from the field, as such reproduction is challenging due to long engine run times, variation in engine operation, and the highly complex fuel mixtures resulting from refilling of the tank. Instead, this study focused on producing and characterizing deposits from a coolant-fuel mixture in order to gain understanding of which types of coolant components contribute to deposits and how such deposits can form. A second aim of the study was to identify potential markers of coolant contamination in IDIDs, thereby improving root cause analysis of failed injectors.

## Methodology

### 1.1 Materials

A failed injector from the field (Europe), where the needle had been stuck in the sleeve has been analyzed. This injector came from a fuel system that was known to have experienced coolant contamination after more than 100,000 km of operation. Unfortunately, neither the extent nor the duration of the contamination was known – only that coolant was found in the fuel system, the fuel filter that had been installed did not have the capability to remove water from the fuel, and that the injector had failed.

The test fuel used in laboratory experiments was B7, a commercial diesel blend where the biodiesel component was rapeseed methyl ester (RME), with coolant added at concentrations of 0.1 and 0.5 wt %. The properties of the B7 used in this study are listed in Table S1 in the Supporting Information. The coolant was a mixture of equal volumes deionized water and coolant concentrate, which was a commercial European organic acid technology (OAT) product with a proprietary composition (ethylene glycol ≥ 95 wt %) that is nitrate-free and phosphate-free. The low solubility of coolant in B7 resulted in a solution where the coolant was suspended as small droplets in the fuel mixture. The coolant concentrations were selected to generate sufficient deposits during the test while maintaining a stable dispersion. Coolant concentrations greater than 0.5 wt % were found to be difficult to run in the test rig reproducibly due to phase separation in the feed tank, which resulted in inconsistent test fuel compositions. The feed container was stirred during the duration of each test to ensure a consistent mixture and that no phase separation was observed in the tubing to or from the test rig.

The field and laboratory deposits were rinsed with heptane to remove residual fuel prior to analysis.

### 1.2 Experimental Setup

The experiments to generate deposits in the laboratory utilized the thermal test deposit (TDT) rig, which was introduced in previous articles by Pach et al.^18,53^ as an effective method for studying IDID formation. This test rig facilitates the formation of deposits from a test fuel on a substrate that can be easily analyzed afterward. The rig consists of a 30 μm-thick piece of aluminum foil where deposits form and a 2 mm thick fluorine Kautschuk material (FKM) gasket sandwiched between two aluminum blocks. The test rig is set on a heating plate. The deposits are formed along a flow path of 130 mm with a temperature gradient of 100 to 200 °C. This gradient is similar to that seen in high-pressure injectors, where the fuel entry point is approximately 100 °C and the temperature at the tip of the injector depends on the engine operation, often being around 280 °C. Although the materials of the test rig are prohibitive for running at higher temperatures, the test rig has been demonstrated to be an effective test method for the study of internal diesel injector deposits.^18,53^ The gradient is generated by cooling water fed through the upstream end of the rig. The test fuel is fed to the rig using a diaphragm pump operating with a backpressure of 1.7 bar. A fixed hot plate temperature of 250 °C and a test fuel feed rate of 2.5 mL/min were used for all tests, where 600 g of test fuel was flowed through the unit over 2.5 h without recirculation (single pass). At the conclusion of each test, the TDT was flushed with nitrogen to remove the test fuel and dry the deposits prior to removal of the aluminum foil from the test rig.

### 1.3 Analytical Techniques

The analytical techniques used in this study were the same as those described by Pach et al.^14–16,18,53^

#### 1.3.1 Visual Inspection

The appearance and nature of the deposits were assessed visually. For the field injector, the main areas of interest were the lower parts of the needle and the nozzle, where deposits tend to be most abundant in failed field injectors.^14–16^ Assessment of the field injector deposits, as well as further analyses, required opening of the injector nozzle, which was done via the breaking method (nozzle opening device) reported earlier.^14–16^

#### 1.3.2 SEM-EDX

Scanning electron microscopy (SEM) coupled with energy dispersive X-ray spectroscopy (EDX) was operated using two modes. First, secondary electron mode (SE2) at a distance of 7–12 mm was used to assess the morphology of the sample. The backscattering electron mode (BSE) was then used to determine the elemental composition of the sample, wherein an accelerating voltage of 10 kV was applied to the sample. Images were also collected using variable pressure mode. Elemental compositions in terms of atomic percentages were estimated using point and map analyses. The standardless method had an error margin of ± 5 wt %.

#### 1.3.3 Pyrolysis GC-MS

Pyrolysis (Py) coupled with gas chromatography – mass spectrometry (GC-MS) was used to identify fragments of the deposits. The pyrolysis was performed using a Pyrola 2000 from Pyrol AB and the GC-MS analysis was conducted on an Agilent 6890 GC coupled with a 5977B GC/MSD. The pyrolysis temperature was 600 °C. Tetramethylammonium hydroxide (TMAH, 10% in deionized water) was used as a methylating agent to stabilize fragments of polar compounds, such as alcohols and acids via conversion to methyl ethers and methyl esters, respectively. The gas chromatography column was a HP-5 ms Ultra Inert cross-linked column, made of (5%-phenyl)-methyl polysiloxane. Peaks were identified via comparison to the National Institute of Standards and Technology (NIST) mass spectral library.

#### 1.3.4 FTIR-ATR

The deposit samples were analyzed with Fourier transform infrared spectroscopy (FTIR) on a diamond plate in attenuated total reflection (ATR) mode. The spectrum resolution was 4 cm^–1^ with 5 scans.

## 2 Results and Discussion

### 2.1 Visual Inspection

Deposits were observed throughout the injector, both on the needle and in the nozzle, including above the guidance, in greater amounts than those normally seen in injectors from the field. These thick deposits on the needle and nozzle were light brown and not sticky. No restrictions were observed in the nozzle holes.

Deposits generated in the laboratory test rig were soft, sticky, and weakly adhered to the aluminum foil. If an excessive nitrogen flow was used upon completion of the test, the deposits could be redispersed toward the outlet. The deposits, which appeared yellowish and lumpy, were visible to the naked eye and were most abundant at surface temperatures of 150 °C or higher. At lower temperatures (110 °C), deposits were present but less apparent. The higher concentration of coolant led to more visible deposits; consequently, analyses were focused on deposits from the test fuel with 0.5 wt % coolant.

### 2.2 SEM-EDX

#### 2.2.1 SEM-EDX of the Field Injector

The deposits in the needle sleeve and on the needle were comprised of three types of deposits: a cracked layer, globular cluster deposits, and particulate deposits.

##### 2.2.1.1 Cracked Layer Deposits

The cracked layer deposits were widespread but most visible further from the injector tip. An example of this type of deposit is shown in Figure 1 and the corresponding elemental composition data in atomic percentage (at. %) are shown in Table 1. This table shows both data from the entire map, as well as from five point analyses. One point, Spectrum 10, was inside a crack, where the deposit is shown to be thin, as seen from the high proportion of iron (injector surface) and low proportions of carbon and oxygen. From the map data and the four other point analyses, the major components of the cracked layer are seen to be carbon, oxygen, sodium, sulfur, and calcium. Calcium and sodium may be in the form of carboxylates, sulfates, or a combination of the two. Phosphorus, magnesium, potassium and aluminum follow a similar dispersion pattern as calcium and sodium, while nitrogen and silicon were not found to be significant components of the cracked layer.

**Figure 1:**
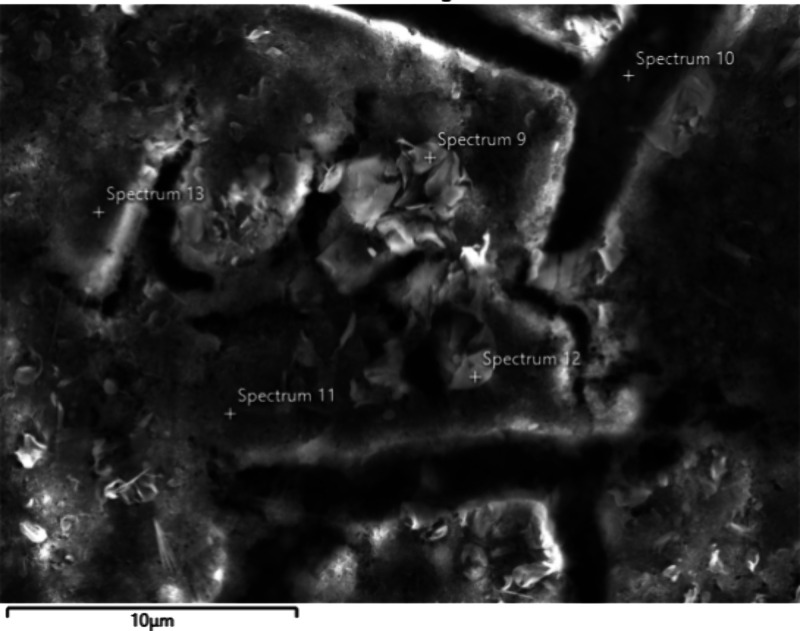
SEM image of deposits on lower sleeve of field injector using vacuum and SE2 detector, away from tip. Magnification 4.08 k X, 10 kV, working distance 11.5 mm.

**Table 1:** Element Map Analysis and Point Analyses for Cracked Layer Field Deposits in Figure 1

element	total map [at. %]	spectrum 9 [at. %]	spectrum 10 [at. %]	spectrum 11 [at. %]	spectrum 12 [at. %]	spectrum 13 [at. %]
carbon	51.1	52.7	10.4	56.4	38.8	55.2
oxygen	27.9	24.5	2.5	24.2	24.8	24.4
sodium	7.0	7.9	1.4	6.1	9.0	7.2
sulfur	5.2	6.5	1.4	5.6	8.6	5.4
calcium	3.4	4.1	0.7	3.9	8.6	3.7
iron	2.3	0.6	81.9	0.5	2.3	0.6
phosphorus	1.2	1.5	0.5	1.4	2.6	1.3
magnesium	0.8	0.9	0.3	0.8	1.7	0.9
potassium	0.7	0.9	0.0	0.8	1.2	0.8
aluminum	0.3	0.3	0.0	0.3	0.7	0.4
silicon	0.0	0.0	0.0	0.0	0.0	0.1

##### 2.2.1.2 Globular Cluster Deposits

Globular cluster deposits were observed in the lower part of the injector on top of a cracked, lower layer along with needle-like crystals, shown in Figure 2. The elemental composition appeared to be similar for both the needle clusters and the larger aggregates. Underneath the globular cluster deposits, there appeared to be a layer with cracks.

**Figure 2:**
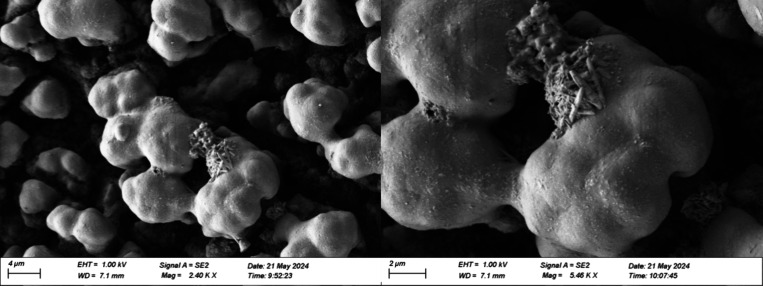
SEM images of deposits on lower sleeve of field injector using vacuum, SE2 detector.

SEM-EDX results shown in Figure 3 suggest that the globular cluster deposits contain mainly sodium carboxylates, as the predominant elements found were carbon, oxygen, and sodium. Nitrogen, aluminum, and magnesium also appeared to be associated with the globular cluster structures but were much less abundant. Sulfur, calcium, phosphorus, and potassium were also found by EDX, but these elements appeared to be widely more dispersed and not associated with the globular cluster structures. Silicon was not observed in significant amounts. Iron is generally associated with the injector itself and is seen at higher levels where the deposits are thinner.

**Figure 3:**
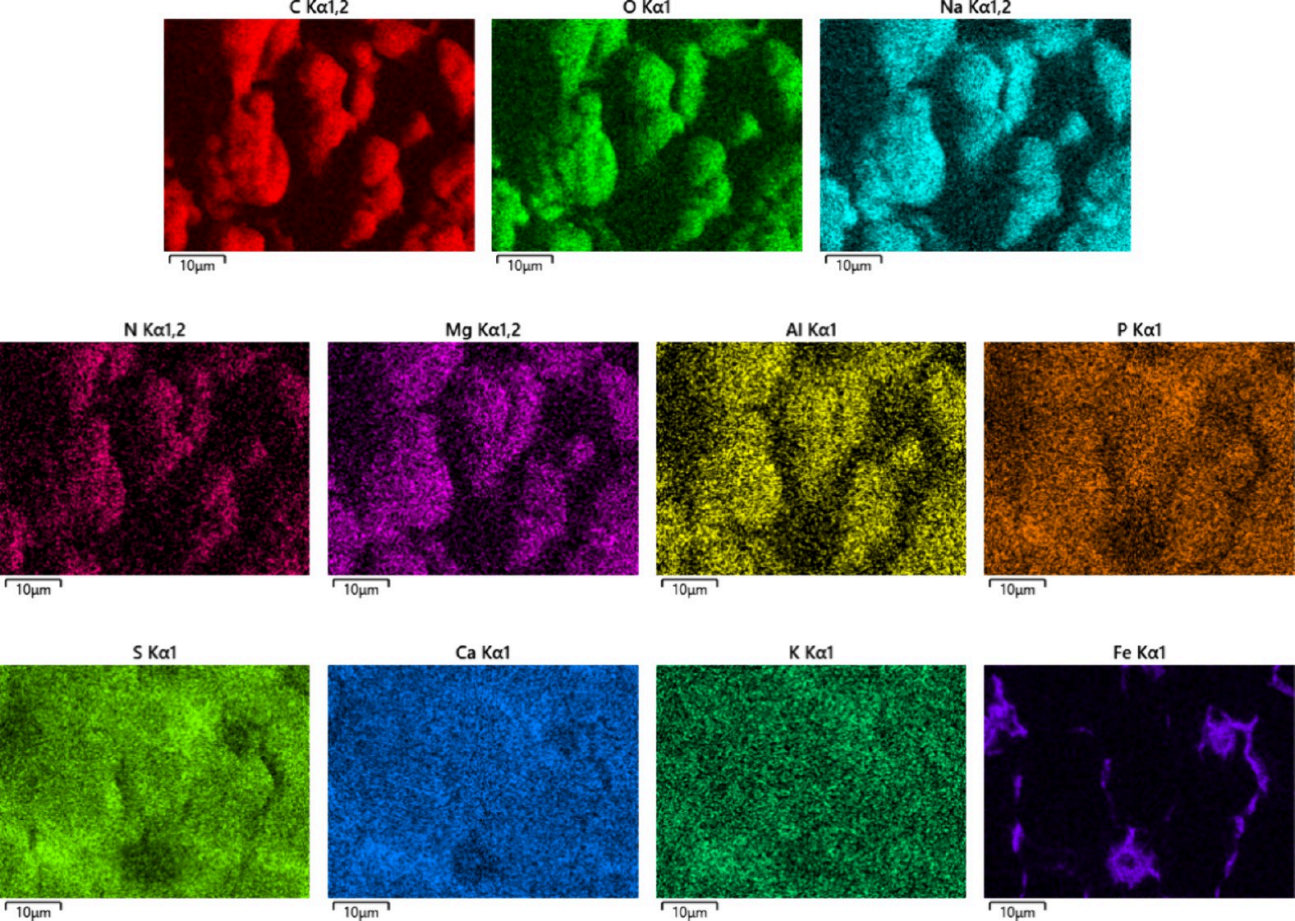
Elemental mapping images of field injector deposits, lower injector sleeve using SEM-EDX.

Table 2 compares the elemental map analysis from SEM-EDX of cracked and globular cluster deposits from the field injector, where the compositions are given in atomic percentage (at. %). The cracked deposits contain greater amounts of sulfate salts and metals than do the globular cluster deposits, which contain more carboxylates, as seen in the relative proportions of carbon and oxygen. Due to the penetrating nature of the EDX analysis, the data for the globular cluster deposits also include the underlying cracked deposits.

**Table 2:** Element Map Analyses Data for Cracked and Globular Cluster Deposits from the Field Injector via SEM-EDX

element	cracked [at. %]	globular cluster [at. %]
carbon	51.1	68.8
oxygen	27.9	17.1
sodium	7.0	2.7
sulfur	5.2	2.5
calcium	3.4	1.9
iron	2.3	2.3
phosphorus	1.2	0.6
magnesium	0.8	0.3
potassium	0.7	0.4
aluminum	0.3	0.2
nitrogen	0.0	3.0
silicon	0.0	0.0

##### 2.2.1.3 Additional Particulate Deposits

The images (Figures 4 and 5) below appear to depict some particulate matter on the deposits that may have originated from an external source, as the composition differs from the surrounding deposits. The EDX analysis of the deposit in Figure 5 is shown in Figure 6.

**Figure 4:**
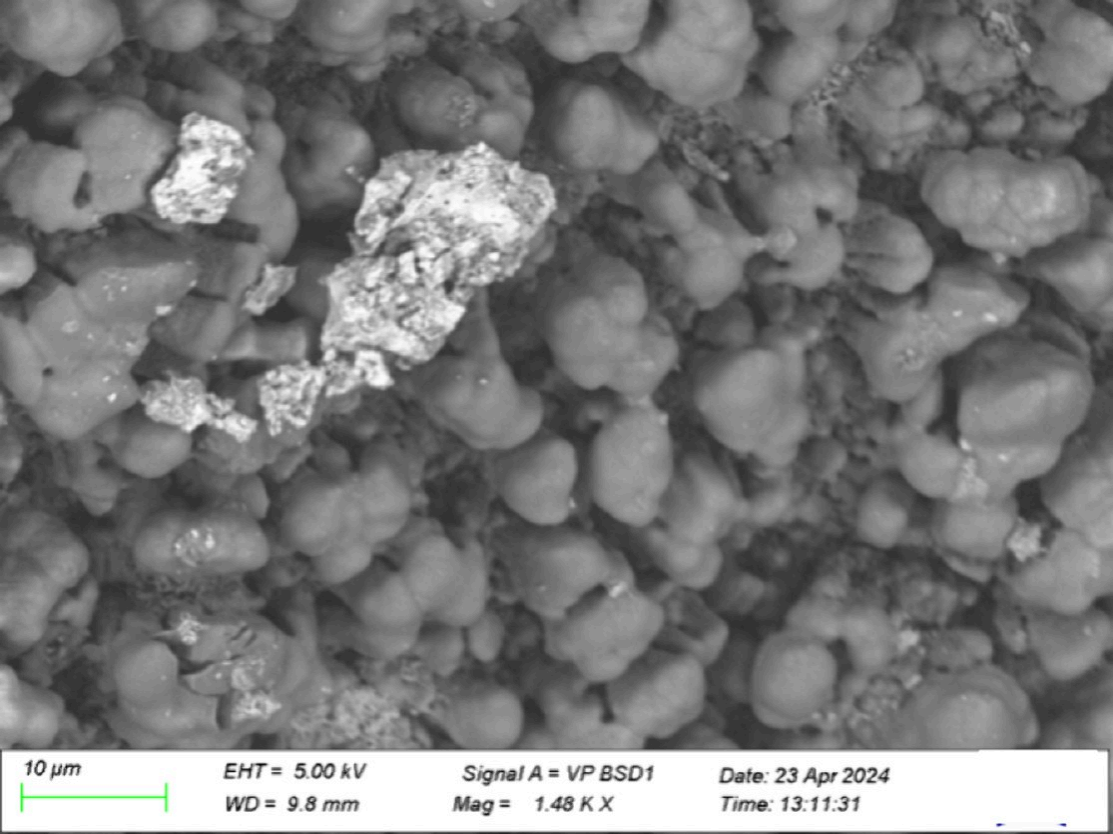
SEM image of field injector deposits with particulates, lower injector sleeve, first location, using variable pressure mode.

**Figure 5:**
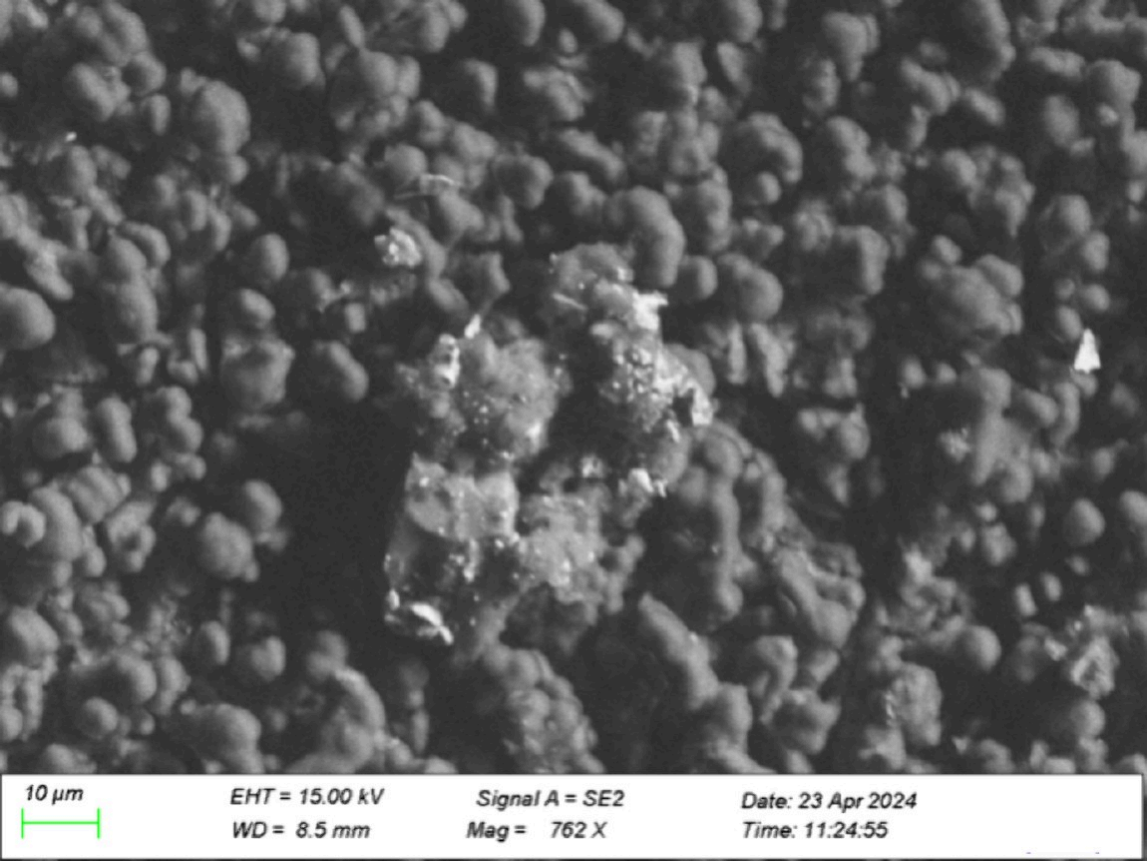
SEM image of field injector deposits with particulates, lower injector sleeve, second location, using vacuum and secondary electron mode.

**Figure 6:**
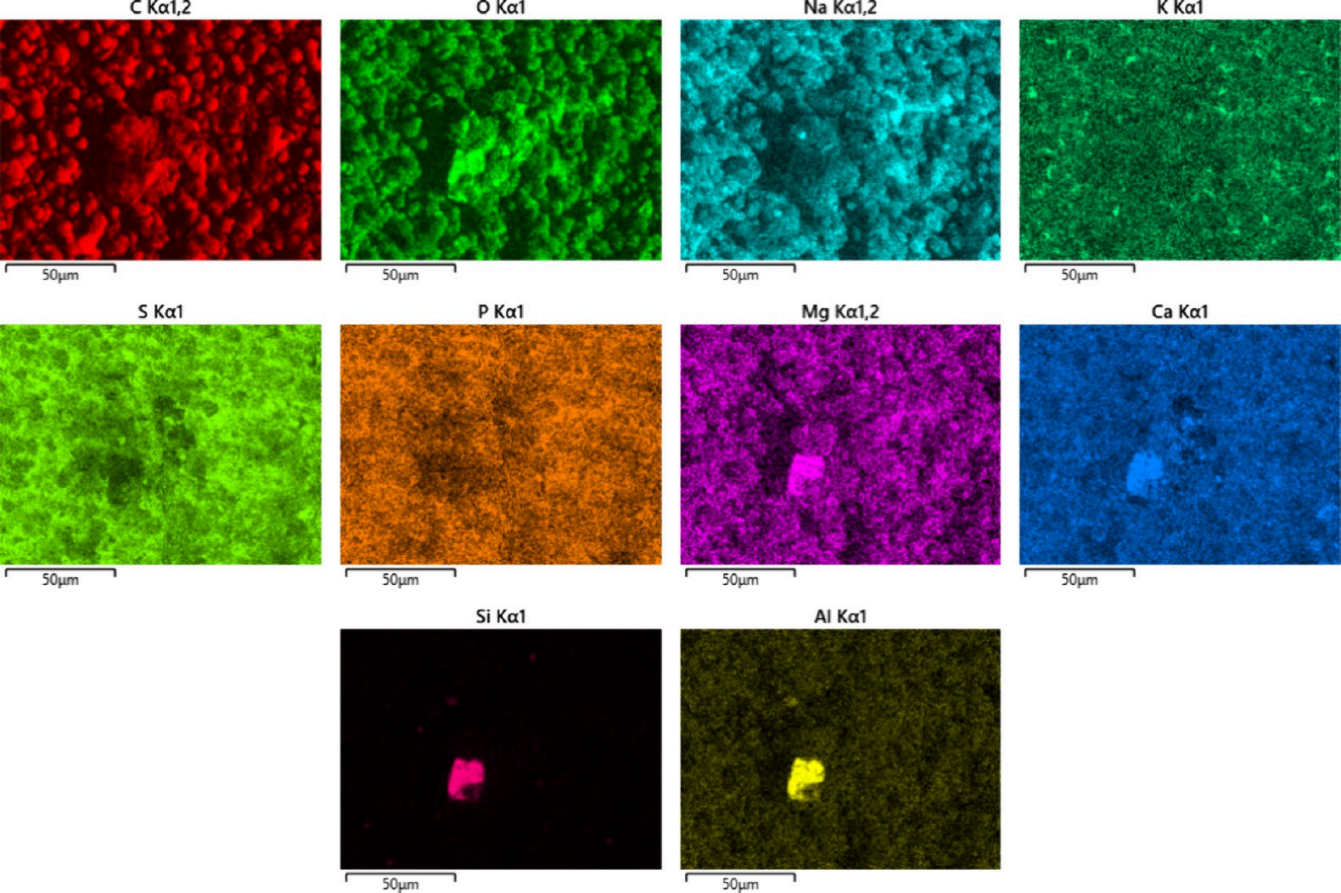
Elemental mapping of field injector deposits using SEM-EDX from Figure 5

#### 2.2.2 SEM-EDX of the Laboratory Deposits

The structures of deposits from the laboratory test rig observed using SEM appeared highly porous, with a “stringy” appearance. These structures are seen in Figure 7. No differences were observed between deposits using 0.1 wt.% coolant and deposits using 0.5 wt.%, so the SEM-EDX and other analyses focused on the latter, as these deposits were greater in abundance. The data from the SEM-EDX, shown in Table 3, suggest that these deposits are primarily sodium carboxylates. A small amount of silicon was also observed, but was dispersed, rather than integrated with the structures of the deposits. The composition of the deposits was consistent over the length of the test rig and between experiments, predominantly sodium carboxylates. For this test rig, the presence of aluminum detected in the EDX results was due to the aluminum foil substrate on which the deposits formed, suggesting the deposits were thin.

**Figure 7:**
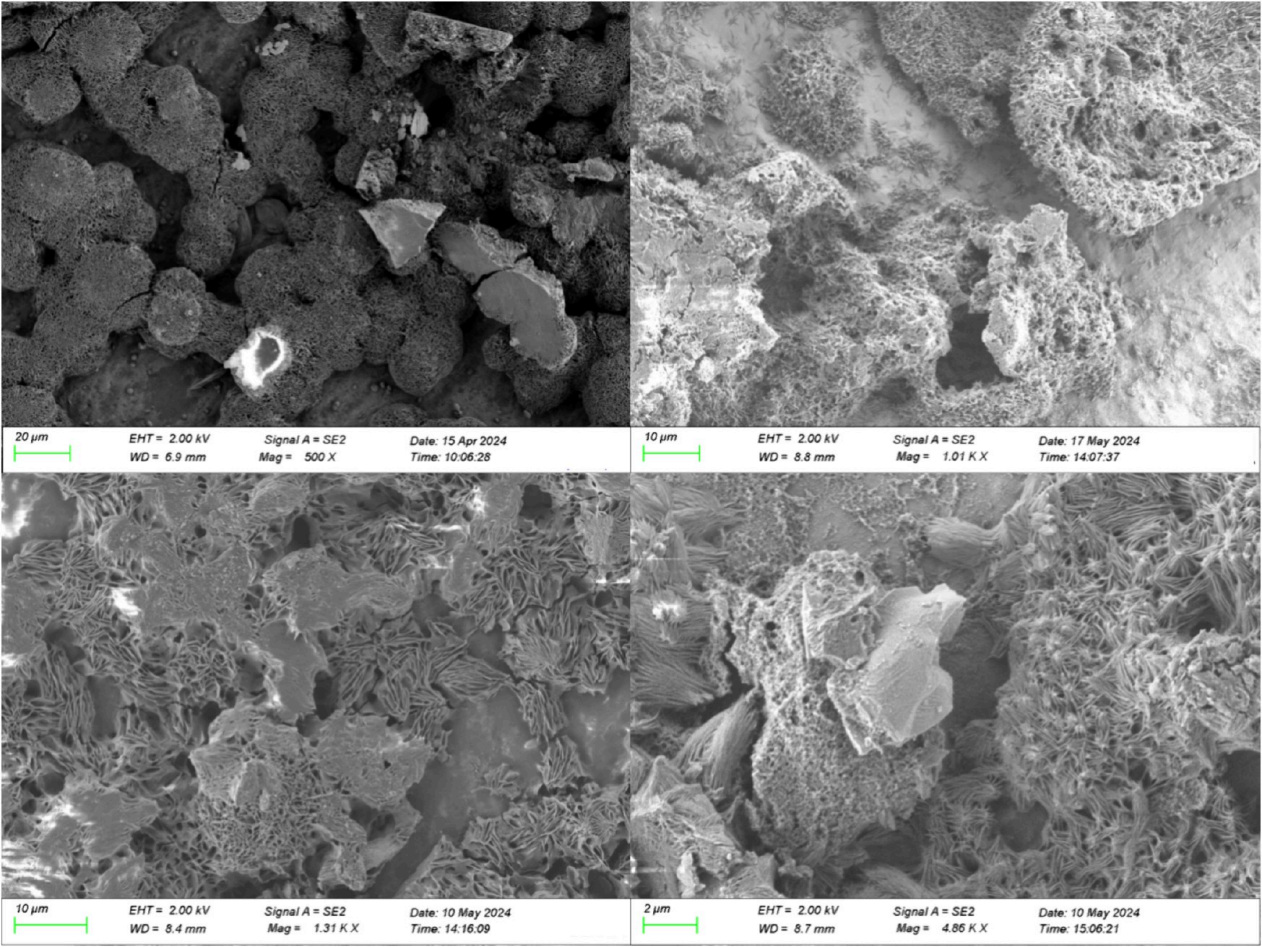
SEM images of deposits from the laboratory test rig using vacuum and secondary electron mode.

**Table 3:** Element Map Analysis of Laboratory Deposits Using SEM-EDX

element	deposits [at. %]
carbon	64.1
aluminum	20.1
oxygen	12.4
sodium	2.9
potassium	0.1
silicon	0.1

The structure of coolant dried at ambient temperature and pressure lacked the stringy or porous structure of the deposits from the test rig and appeared to be more compact, as seen in Figure 8. SEM-EDX analysis of dried coolant, shown in Table 4, revealed carbon, oxygen, and sodium to be the major components and silicon and potassium to be minor components. Since carbon tape was used for the analysis of the dried coolant, the measured carbon content is higher than the actual content. However, the identification of the other elements present and their relative amounts are of greater interest: sodium is a significant component, potassium is present but in an amount on an order of magnitude lower than that of sodium, and silicon is present in an amount below that of potassium.

**Figure 8:**
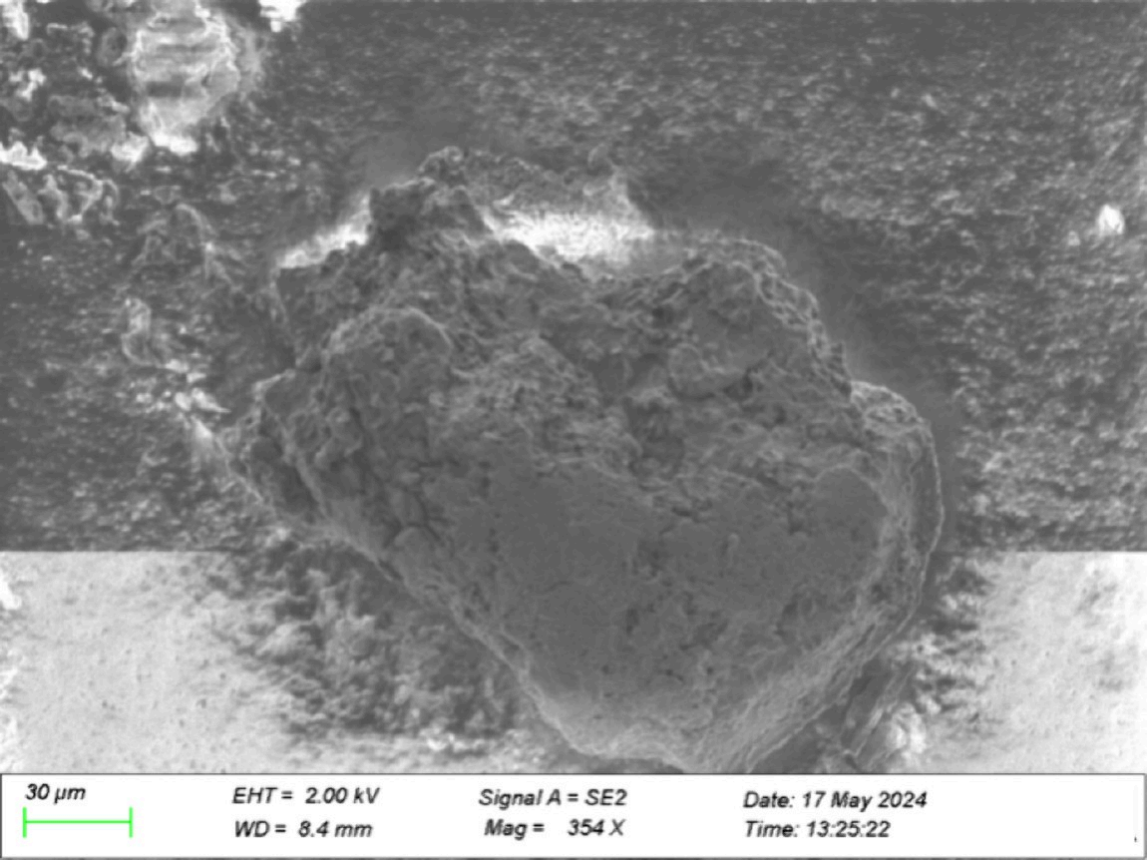
SEM image of dried coolant using secondary electron mode at a magnification of 354 X.

**Table 4:** Element Map Analysis of Dried Coolant on Carbon Tape Using SEM-EDX

element	deposits [at. %]
carbon	69.7
oxygen	22.8
sodium	7.0
potassium	0.3
silicon	0.1

### 2.3 Pyrolysis GC-MS Results

#### 2.3.1 Pyrolysis GC-MS of the Field Injector

The pyrolysis GC-MS data from the field injector, seen in Figure 9 with selected peaks labeled with letters, showed a complex mixture of compounds. These data revealed the presence of sulfate (*c*), phosphate (*e*), 2-methoxybenzoic acid, methyl ester (*i*), dimethyl phthalate (*j*), and 1,4-benzenecarboxylic acid, dimethyl ester (*l*). Tetramethylsilicate (*a*) and disilicic acid (*g*), hexamethyl ester were observed when TMAH was used, consistent with methylation of the silicon components of the pyrolyzed deposit. Derivatives of 1H-benzotriazole (*g* and *k*), a corrosion inhibitor commonly used in coolant formulations, were also found. Other components found include carboxylic acid derivatives, such as dimethyl fumarate and methyl esters of butanedioic acid, aspartic acid, hexadecanoic acid, and octanedioic acid. Interestingly, some of the metal carboxylates commonly seen in field fuel systems, such as decanedioate and nonanedioate,^36^ were not observed in significant amounts. A variety of nitrogen-containing species that are not solely attributable to side reactions with TMAH (e.g., trimethylamine and glycine, *N,N*-dimethyl-, methyl ester – *b* and *d*) were also observed but not definitively identified.

**Figure 9:**
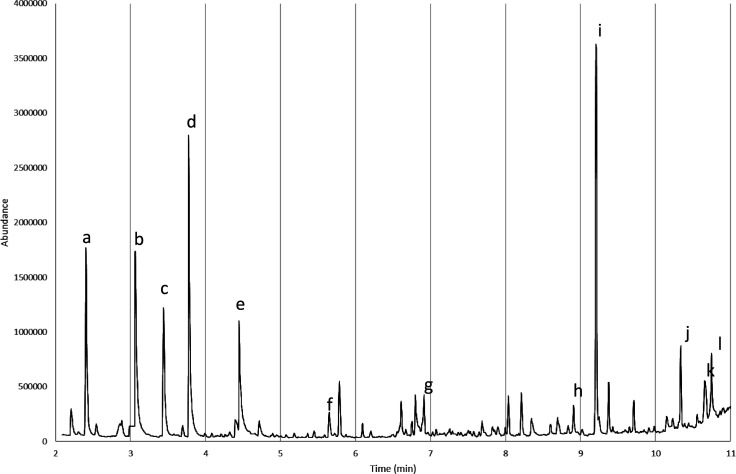
Highlighted pyrolysis GC-MS results from the field injector deposit.

#### 2.3.2 Pyrolysis GC-MS of the Laboratory Deposits

The most predominant component of the deposits from the laboratory test rig was decanedioic acid (C_10_), found as the dimethyl ester when using TMAH in pyrolysis GC-MS. Other acids found as methyl esters were hexanedioic acid (C_6_), methyldecanedioic acid (C_11_), dodecanedioic acid (C_12_), octadecenoic acid (C_18_), and eicosadienoic acid (C_20_). Dibutyl phthalate was another component found in the laboratory deposits via pyrolysis GC-MS. As this ester was a butyl ester, not a methyl ester, it can be concluded that this ester did not form from a reaction with TMAH. Silicate, sulfate, and phosphate were not observed in the analysis of the laboratory deposits.

### 2.4 FTIR-ATR

#### 2.4.1 FTIR-ATR of the Field Injector

A portion of the FTIR-ATR spectrum (1850–650 cm^–1^) from the field injector is shown in Figure 10, while the full infrared spectrum can be found in the Supporting Information.

**Figure 10:**
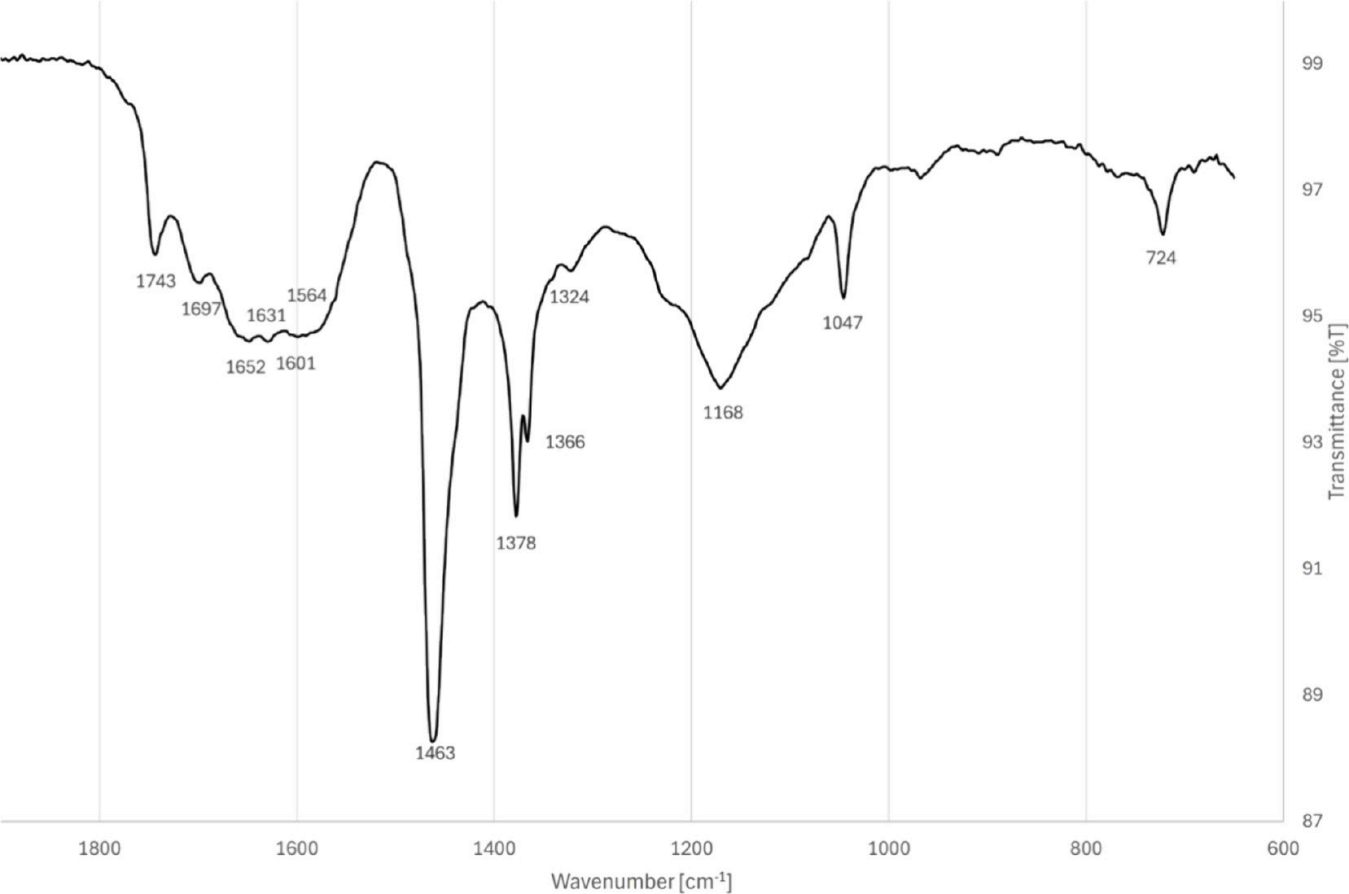
FTIR-ATR spectrum of field injector deposits.

There are some peaks in common with B7, such as those at 1463, 1378, and 724 cm^–1^; it is not unusual for traces of fuel to be observed when analyzing deposits. Aged biodiesel could also be present, as the FAME 1747 cm^–1^ broadens and shifts toward lower wavenumbers (1740 cm^–1^) during autoxidation. Peaks associated with C–O linkages around 1100 cm^–1^ and carbonyls at 1697 cm^–1^ have also been reported to increase with the aging of biodiesel.^54^

The FTIR-ATR data shows similarities to those of filterable, insoluble diesel gums generated by accelerated oxidation of B0 reported by A.J. Power, with large, broad peaks around 1610 and 1150 cm^–1^, as well as peaks around 1460 cm^–1^.^55^ Power hypothesized that these insoluble gums may have been aromatic nuclei cross-linked by ether bridges, since the band at 1610 cm^–1^ was attributed to aryl C = C­(-O) stretching vibrations and the strong absorptions between 1300 and 1050 cm^–1^ were attributed to aromatic ether moieties. Similarly, Gopalan et al. analyzed insolubles from aged B0 and B10 and attributed peaks between 1525 and 1607 cm^–1^ to aromatic carbon bonds.^56^ Pyrolysis GC-MS revealed 2-hydroxybenzoic acid (salicylic acid) in the deposits, which is believed to originate from the coolant and may have reacted and formed more complex structures, potentially resulting in the FTIR-ATR spectra with similarities to those reported by Power.

Carboxylate bands generally are reported in the ranges of 1575–1530 and 1460–1400 cm^–1^, where the exact locations of the carboxylate peaks depend on the cation and the type of coordination with the cation. Sodium carboxylates have been reported to have peaks around 1560 cm^–1^; for example, sodium stearate has an asymmetric doublet at 1573 and 1559 cm^–1^.^57^ Bands for CH_2_ absorptions are seen at 1400–1100 cm^–1^ and 721 cm^–1^ for sodium and calcium carboxylates.^57^ Sodium carboxylates clogging fuel filters have been reported in the literature with peaks at 1562 and 1460 cm^–1^.^47^ Coordination with water also affects peak locations; for example, hydrated calcium salts have broad peaks around 1630 and 3440 cm^–1^.^57^

Sulfates and phosphates have peaks around 1100 cm^–1^.^15,16,18^ The location of sulfate peaks depends on the hydration state and the local environment, where the dihydrate absorbs at higher wavenumbers than the hemihydrate.^58^ The broad peak from 1250 to 1080 cm^–1^ seen for the field injector in Figure 10 could include contributions from the sulfates and phosphates that were observed in the Py GC-MS.

It appears that acids may also be present, in addition to carboxylates. A diacid carbonyl peak is reported around 1695 cm^–1^, as mentioned earlier with aged biodiesel. Crystallization of fatty acids together with their sodium salts have been reported to have carbonyl peaks around 1740 cm^–1^.^57^

Similarities can be seen to field deposits from a vehicle with an oil-lubricated high-pressure pump (HPP) operating on FAME reported previously by Pach et al.: a large carboxylate peak in the range of 1700–1550 cm^–1^.^14,15^ This region is notoriously difficult to interpret, however, due to the various possibilities for peaks, including amides and amines.^15^

#### 2.4.2 FTIR-ATR of Deposits from the Laboratory Test Rig

The FTIR spectrum of the laboratory deposits, shown in Figure 11 and together with the spectrum of the field deposits in Figure 12, are consistent with a combination of mono- and disodium salts. The major peaks at 1558, 1443, and 1415 cm^–1^ are aligned with those of sodium carboxylate salts.^29,59^ Specifically, monosodium carboxylates are consistent with peaks at 1558 and 1425 cm^–1^, whereas sodium decanedioate (identified in the laboratory deposits via pyrolysis GC-MS) has been reported to have peaks at 1563 and 1416 cm^–1^.^60^ Minimal water and ethylene glycol were observed in the laboratory deposits, as the peak around 3300 cm^–1^ is largely absent.

**Figure 11:**
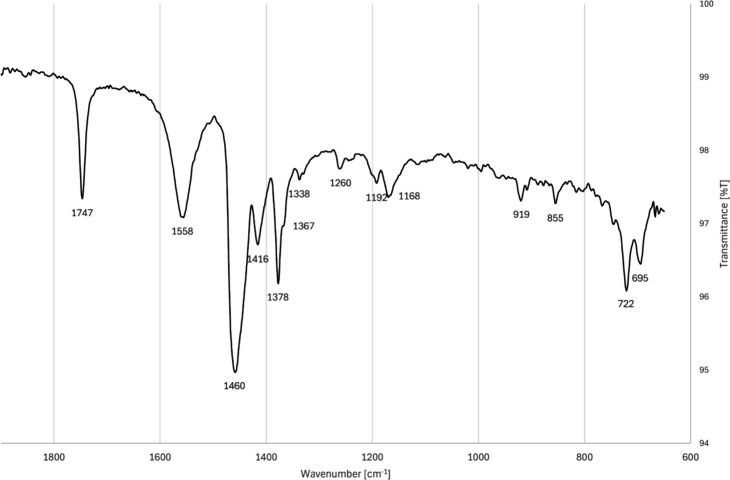
FTIR-ATR spectrum of deposits from the laboratory test rig

**Figure 12:**
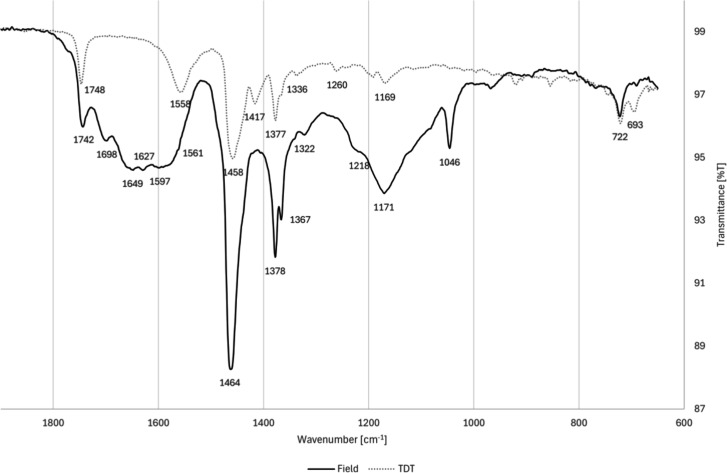
FTIR-ATR spectrum of laboratory deposits (dashed line, TDT) and field injector deposits (solid line).

The primary similarities between the laboratory and the field injector FTIR spectra, shown in Figure 12, are due to the predominance of sodium carboxylates. The deposits differ in metal carboxylate composition – both in the carboxylate speciation and the lack of calcium in the laboratory deposits. Other differences include the presence of sulfates and phosphates in the field injector deposits but not in the laboratory deposits, contributing to a simpler spectrum for the laboratory deposits.

### 2.5 Discussion

#### 2.5.1 Deposit Composition

The field deposits were found to contain predominantly sodium carboxylates as well as a variety of other components in lesser amounts. Free ethylene glycol was not seen as a significant component of the field deposits, based on the FTIR-ATR results. Deposits from the field injector were found to contain low-carbon-number carboxylic acid derivatives, including dimethyl fumarate, butanedioic acid esters, and oxalic acid mono-(*N*-dimethyl)-amide, methyl ester when analyzed using TMAH. A possible origin of these compounds could be ethylene glycol degradation, which has been reported to produce formic acid, oxalic acid, acetic acid, and glycolic acid.^61–63^ It is possible that some ethylene glycol could have reacted with free fatty acids or these degradation products to form esters.

Sodium, commonly included in coolant as sodium silicate, was seen in both the laboratory and the field deposits. While sodium contamination of biodiesel blends is not uncommon,^11^ the amounts seen in these deposits were high (current B100 limits are 5 ppm of Na)^4^ and were in line with literature and IDIDs from the field.^31,47^ Since metal content has been reported to be the limiting parameter for metal carboxylate deposit formation due to the amounts of fatty acids normally present,^32^ the high sodium content is a key contributor to these deposits.

Silicon is sometimes seen in diesel from antifoaming agents, but typically at low ppm levels. Silicates were observed in the field injector deposits using pyrolysis GC-MS. Gel formation has been reported in coolant systems when the silicate encounters acid. Silica gel can form via the hydrolysis of silicate (Na_2_SiO_3_) to silicic acid (Si­(OH)_4_), followed by polymerization.^64^ While silicate deposits resulting from polymerization and gelation are known to occur in coolant systems,^65^ such deposits were neither observed, neither in the field, nor in the laboratory. In both cases, silicon was observed using SEM-EDX to be dispersed rather than associated with the globular cluster deposit structures. Additionally, some particulates containing silicon were observed in the field injector that appeared to have formed elsewhere and subsequently settled on the surface of the IDIDs.

Potassium was seen in the field injector deposits but not in the laboratory deposits. Potassium contamination could originate from contaminated biodiesel or from carboxylate additives in the coolant, depending on the formulation. The potassium observed using SEM-EDX was generally dispersed but some particles containing potassium were found along with silicon, and aluminum. The combination of these elements suggests coolant as the source, wherein aluminum can come from corrosion of the cooling system. It is possible that the coolant that contaminated the fuel for the field injector contained potassium while the coolant used in the laboratory experiments did not.

Nitrogen was observed in the globular cluster and particulate deposits but not in the cracked layer deposits. The nitrogen-containing particulates observed in the SEM-EDX analysis did not appear to contain significant amounts of sulfur, suggesting that the nitrogen was not present as benzothiazole but rather as another species, such as a benzotriazole or other metal deactivator or corrosion inhibitor. Such additives are used to form complexes with metal ions, consistent with the observed association of aluminum with nitrogen in a particulate deposit. A variety of nitrogen compounds in the field deposits that were observed but not identified in the pyrolysis GC-MS data could have originated from metal deactivators or corrosion inhibitors, such as triazoles from coolant^66^ or *N,N*’-disalicylidene-1,2-propanediamine from fuel.^67^ The pyrolysis GC-MS results appear to indicate the presence of benzotriazole derivatives. Bittering agents used in coolant (denatonium benzoate)^68^ also contain nitrogen and could have been incorporated in the field deposits at low levels.

Sulfates were detected in the field deposits using Py GC-MS. It is possible that this resulted from the reaction of TMAH with sodium or calcium sulfate and subsequent decomposition during pyrolysis. Sulfates in IDIDs are primarily associated with oil contamination of the fuel. Sulfur compounds remaining in diesel after hydrotreatment are typically refractory benzothiophenes that are not readily converted to sulfates.^69^ The presence of ethylene glycol in water is known to reduce the solubility of sulfate salts to such an extent that it has been deemed an antisolvent.^70^ Consequently, the presence of ethylene glycol in diesel could potentially accelerate inorganic salt fouling due to decreased solubility in water droplets suspended in the fuel. Sulfur may be present in certain coolant formulations as benzothiazoles for corrosion inhibition. However, the presence of sulfur observed in the SEM-EDX analyses being primarily in the cracked layer, as seen in Table 1, is more consistent with typical, oil-related deposits, resembling IDIDs previously reported in the literature from vehicles with oil-lubricated high-pressure pumps that leak into the fuel system.^14–16,18^

Calcium was observed in the field deposits, with higher amounts seen in the cracked layer than in the globular cluster or particulate deposits. In the laboratory deposits, however, calcium was not observed. This was expected since calcium is not a typical component of coolant. The most common origin of calcium in injector deposits is engine oil but it can also originate from poor quality biodiesel.^71^ Magnesium was also detected by SEM-EDX in some locations in the field injector but not in the laboratory deposits. Magnesium has previously been observed in field IDIDs^59,72^ and can be associated with poor quality biodiesel^32,71^ or engine oil contamination.^16^

Phosphate, seen in the field deposits but not the laboratory deposits, can originate from oil additives or from buffers in the coolant. However, phosphate is no longer used in coolants in Europe and phosphate-containing coolants generally do not contain silicate. It is more likely that the phosphates seen in the field deposits originated from oil contamination rather than from coolant contamination, due to the distribution of phosphorus primarily in the cracked deposits rather than in the globular cluster deposits. The presence of sulfur furthers the hypothesis that oil contamination may have been a factor in the formation of field deposits.

#### 2.5.2 Deposit Morphology

Diesel gum particles have been reported in the literature as spheres with diameters ranging from 0.2 to 2.5 μm.^73^ These gums consist of a combination of degradation, oxidation, and polymerization products, having both fuel-soluble and water-soluble moieties, including esters and ethers. When water is present, gum particles are known to cluster as flocs.^73^ Autoxidation often plays a significant role in the formation of injector deposits and deposits resulting from agglomeration of such spherical particles have been reported in the literature.^74^ The SEM images of the field deposits (particularly Figure 2) showed that the globular cluster deposits were composed of aggregates of particles, suggesting that similar phenomena could be at play. It is possible that coolant contamination increased the rate of autoxidation of the fuel, since the polar environment of the coolant or coolant droplets suspended in the fuel could stabilize radical intermediates.^37,71,75,76^

Cracked layers are common in diesel injector deposits, particularly with thick, aged deposits^77^ and with metal carboxylate deposits.^14,16,26^ Metal carboxylate injector deposits are known to have weak adhesion to the injector surface, which may increase cracking as the deposits age.^26,72,78^ The cracks in the lower deposit layer likely form upon drying of the deposits.

It is possible that the cracked layer seen in the field deposits (as can be seen in Figure 3, for example) was a rather typical deposit that formed prior to the coolant-related deposits, since the vehicle had been in operation for thousands of kilometers prior to the injector failure. The presence of components usually associated with engine oil contamination, such as sulfates and phosphates are consistent with this hypothesis.^14–16^

The unusual structures observed in the deposits from the laboratory differ from those normally seen for metal carboxylates, including the field injector deposits. There are multiple factors related to the presence of coolant that may explain why these structures were seen here but not elsewhere: carboxylate composition, high sodium concentration, additional chemical compounds (e.g., hydroxybenzoic acid, phthalic acid), and the nature of the laboratory test rig (slow cooling rates, no complex aging cycles). The structures of the field deposits are also subjected to a complex aging history related to engine operation, involving fluctuations in temperature and fuel flow. An additional factor could be the coolant concentration, which was unknown in the field injector.

The porous structure of the laboratory deposits was unusual and does not appear to have been previously reported in the literature for fuel deposits. However, the structure has similarities to other reports in the literature, for example, the soap structure seen in greases. The structure of soap used as a thickener in grease is made of soap fibers that have been observed in SEM images.^79–86^ The majority of soap-thickened grease produced today uses 12-hydroxystearic acid along with lithium hydroxide to form lithium 12-hydroxystearate. Other fatty acids can also be used, even vegetable oil fatty acids along with soybean oil as the base oil that serves as the solvent for the fatty acid and the resulting grease soap.^80^ Some greases have an additional acid salt and are known as complex greases. Acids used in complex greases include inorganic acids (e.g., boric acid and phosphoric acid), short-chain carboxylic acids (e.g., acetic acid and benzoic acid), and dicarboxylic acids (e.g., nonanedioic acid, decanedioic acid, hexanedioic acid, and terephthalic acid).^86,87^ There is considerable overlap between the carboxylic acids used in grease soap formulations and the carboxylic acids used in OAT formulations for coolant. Decanedioic acid is one such example and was found to be the primary component of the laboratory deposits.

The soap structure in grease has been characterized as a crystalline waxy phase.^88–90^ Soap structures vary in the length and width of the soap fibers that form from aggregated micelles dispersed in the base oil. Delgado et al. reported that differences in rheological properties were mainly due to the soap concentration in the reaction mixture, the waxy soap transition at higher temperatures, and the cooling steps.^82,85^ Other factors include the viscosity of the base oil, the solubility of the soap in the base oil, antioxidants or other additives present in the base oil, and the cation of the soap.^82,83,91^

Reversible crystalline structures containing polar structures in deposits formed from aged biodiesel blends were reported by Engeländer et al., who used differential scanning calorimetry to identify a melting point between 70 and 80 °C.^92^ The study did not include SEM imaging, however. While the laboratory experiments in the current study did not include fuel aging, this suggests that the field injector deposits likely contained a combination of biodiesel aging products and OAT components.

A study by Jafari Ansaroudi et al. included an SEM image of paraffin wax precipitated from kerosene (p. 650)^93^ that is strikingly similar to an SEM image of the laboratory deposits from this study, shown in Figure 13.

**Figure 13:**
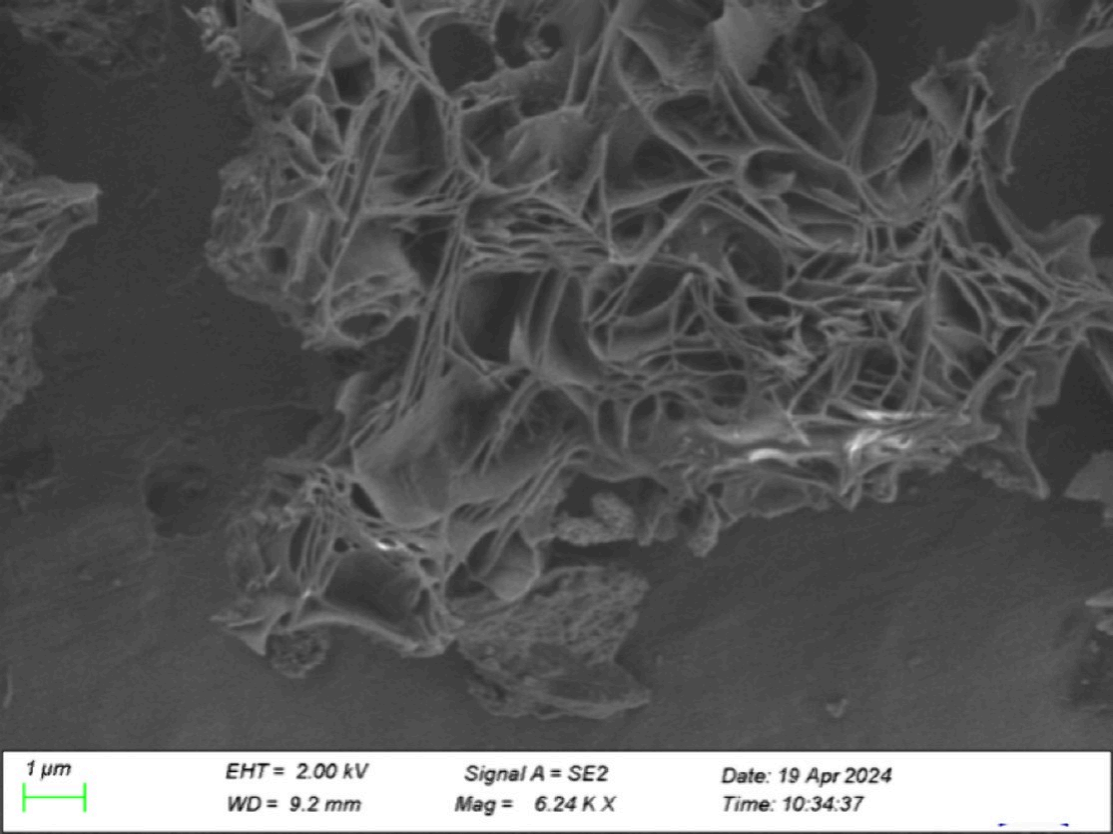
SEM image of TDT deposits using vacuum and secondary electron mode at high magnification (6.24k x).

The structure seen in Figure 13 also has similarities to SEM images of wax-based oleogels reported in the literature.^94^ These similarities in structure suggest that the same underlying phenomena are at work, where plate-like crystals form at lower levels of supersaturation and spherulite-type crystals form at higher levels of supersaturation.^94–96^

## 3 Conclusions

The deposits in the field injector were found to be predominantly calcium, sodium, sulfates, and carboxylates. These deposits were observed to have layers, where sulfate salts were primarily seen in the cracked layer, and the globular cluster layer was primarily composed of metal carboxylates. The characteristics and composition of the cracked layer shared similarities with other documented injector deposits, suggesting that the deposits formed not only due to coolant contamination, but also due to fuel autoxidation and other contamination. For example, the high amounts of calcium and sulfate and lesser amounts of phosphorus and magnesium seen in the cracked layer are consistent with injector deposits from fuel contaminated with engine oil. This suggests that the cracked layer was mainly formed as a typical deposit prior to coolant contamination, while formation of the globular cluster deposit layer was associated with coolant contamination. This conclusion was supported by the finding that both the laboratory deposits and the globular cluster layer were composed predominantly of sodium carboxylates. Silicon is a key component of some coolant formulations, but whereas coolant systems can have silica gelation problems, gelation of silica was not found to be a concern for coolant contamination of fuel, based upon the analyses of deposits from the laboratory rig and the field injector. Ethylene glycol from the coolant was not seen to be a major component of the deposits but is a likely contributor to instability of the fuel mixture via the formation of acids. The presence of water likely also favored deposit formation via increased formation of inverse micelles and/or increased aggregation of particles.

Unique structures were observed in the deposits from the test rig that were unlike the typical structures observed for metal carboxylate injector deposits. It is important to note that the laboratory deposits originated from a simple model system, without any influence from engine oil contamination, aged fuel, and aged coolant in this study. Additionally, the deposits were not subjected to a complex aging process that could alter the structure. The metal carboxylate deposits seen in the laboratory test rig appeared to be similar in appearance to the types of crystalline structures seen in grease soaps, oleogels, and paraffin waxes, suggesting the same phenomena are at work.

Due to the differences among coolants on the market, it is challenging to come up with clear identifiers in injector deposits that indicate coolant contamination of the fuel system. However, in this case, certain components were observed in the deposits that could be considered potential markers for coolant contamination of fuel. The high amounts of sodium seen in the deposits are the main feature of the deposits. Additionally, the significant peaks of hydroxybenzoic acid, terephthalic acid, and silicates seen in the pyrolysis GC-MS results could be considered as signs of coolant contamination, as these are atypical for IDIDs in Europe. Additionally, potassium could also possibly be indicative of coolant contamination of fuel, based on the use of potassium carboxylates in certain coolant formulations and on particles found containing silicon, aluminum, and potassium. While metal carboxylates are a common component of injector deposits, those seen in the field injector were composed of atypical carboxylates: fewer long-chain fatty acid derivatives and more short-chain acids. Furthermore, the deposits seen in the field injector also contained degraded fuel, suggesting that the contamination of fuel with coolant may have accelerated autoxidation of the fuel. The particulate deposits are another potential identifier of coolant contamination in the injector deposits, as these particulates containing aluminum and silicon or aluminum and nitrogen appeared to originate from coolant components.

## Supplementary Material



## Notes

Additional experimental materials data, full FTIR-ATR spectra, and additional SEM images (PDF)

## List of abbreviations

B0: conventional (fossil) diesel
B7: biodiesel blend containing 7 vol % FAME with specifications according to EN 590.
BSE: backscattering electron mode (SEM-EDX)
FAME: fatty acid methyl ester
FTIR-ATR: Fourier transform infrared spectroscopy (FTIR) in attenuated total reflection (ATR) mode
IDID: internal diesel injector deposit
OAT: organic acid technology (coolant formulations)
Py GC-MS: pyrolysis coupled with gas chromatography–mass spectrometry
SE2: secondary electron mode (SEM)
SEM-EDX: scanning electron microscopy with energy dispersive X-ray spectroscopy
TDT: thermal deposit test – laboratory rig
TMAH: tetramethylammonium hydroxide
WD: working distance (SEM)
