# Supplementary material for: An Investigation into Coolant-Related Internal Diesel Injector Deposits from Heavy-Duty Vehicles
Source: ACS Omega. 2025 Jun 4;10(23):24235–51. doi: 10.1021/acsomega.4c11346 (PMC12177768; doi:10.1021/acsomega.4c11346)
Supplement: Supplementary file 1 [file ao4c11346_si_001.pdf]

Supporting information for

An investigation into coolant-related internal diesel injector deposits from heavy-duty vehicles

Sarah L. Hruby[a*], Pavlos Chrysafis[a], Henrik Kusar[a*], Mayte Pach[b], and Henrik Hittig[b]

[a] Department of Chemical Engineering, KTH Royal Institute of Technology, Brinellvägen 8, 114 28 Stockholm, Sweden

[b] Scania Technical Centre, Scania CV AB, Granparksvägen 10, 151 48 Södertälje, Sweden

*Corresponding authors: hruby@kth.se (Sarah L. Hruby) and kusar@kth.se (Henrik Kusar)

Table S1. Properties of the B7 diesel used in the experiments

Figure S1. SEM images of deposits on lower sleeve of field injector using variable pressure mode

Figure S2. Full FTIR-ATR spectrum of deposits from the field injector

Figure S3. Full FTIR-ATR spectrum of deposits from the laboratory test rig

Table S1. Properties of the B7 diesel used in the experiments

Property [units]	Method	Result
Appearance at 20 °C [-]	Visual inspection	Clear and bright
Density [kg/m^3]	EN ISO 12185	836.5
Viscosity at 40 °C [mm^2/s]	EN ISO 3104	2.852
Strong acid number, method A [mg KOH/g]	ASTM D974	< 1.0
FAME content [% v/v]	EN 14078	6.2
Flash point [°C]	EN ISO 2719	68.0
Total contamination [mg/kg]	EN 12662	< 24
Water content [mg/kg]	EN ISO 12937	< 30
Zinc content [mg/kg]	ASTM D6443	< 1.0
Sulfur content [mg/kg]	EN ISO 20846	7.0
Copper content [mg/kg]	ASTM D6443	< 1.0
Ash content [% m/m]	EN ISO 6245	< 0.010

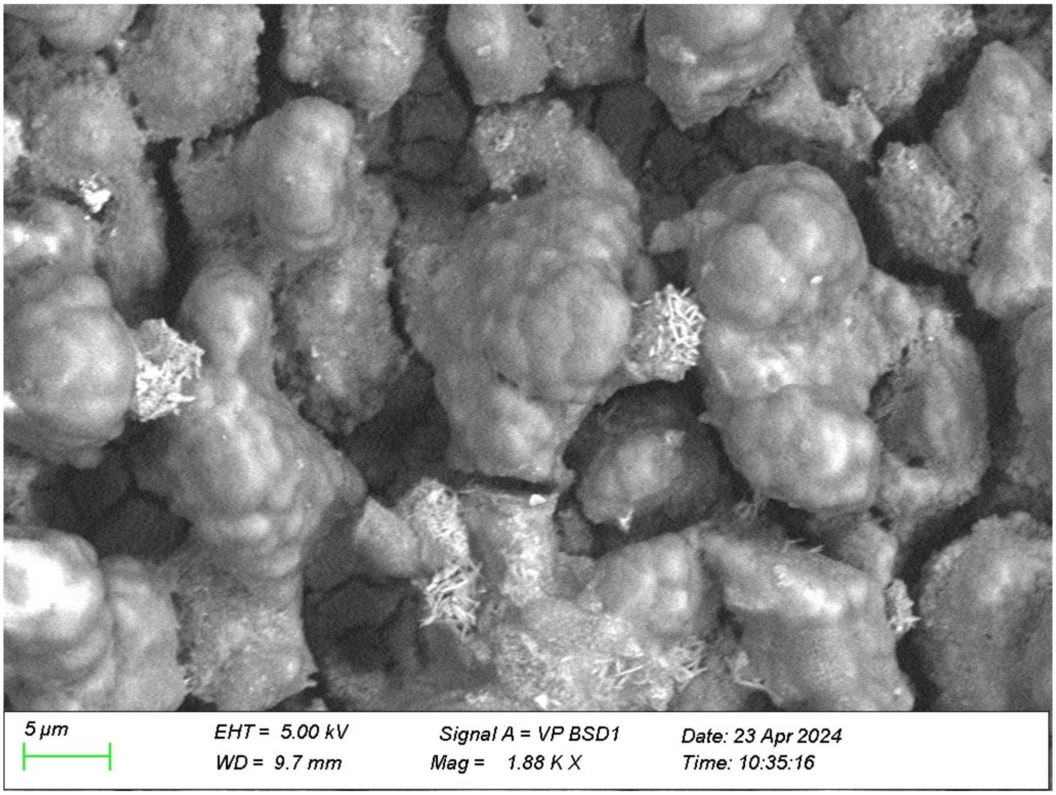

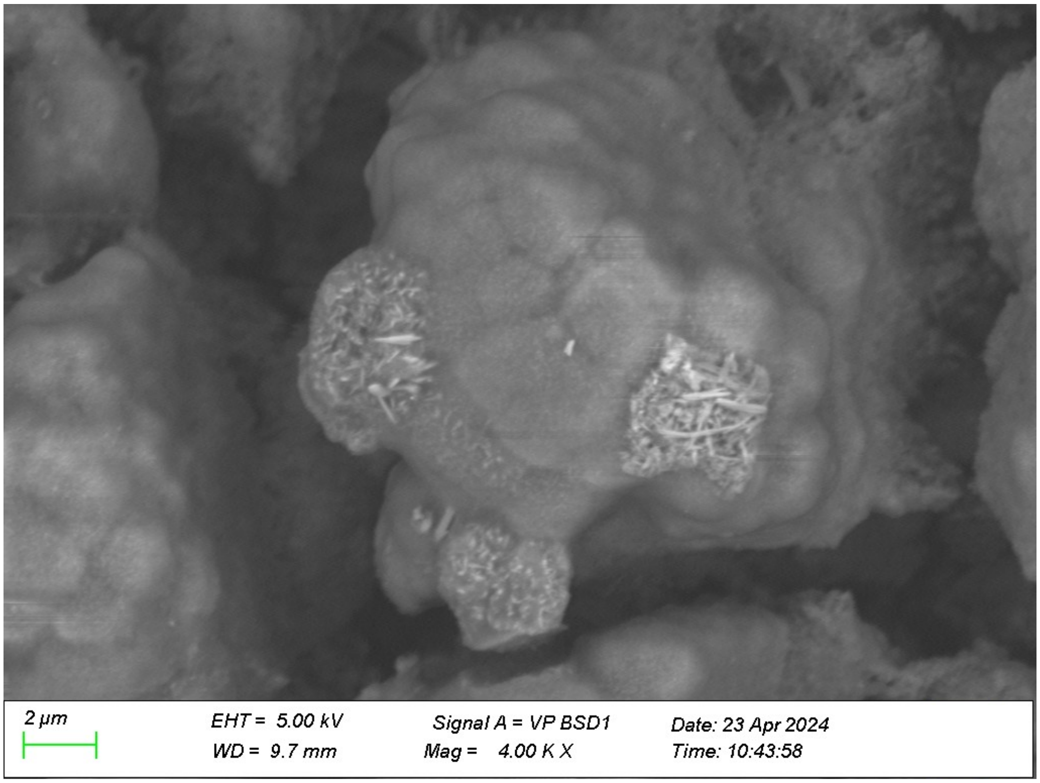

Figure S1. SEM images of deposits on lower sleeve of field injector using variable pressure mode

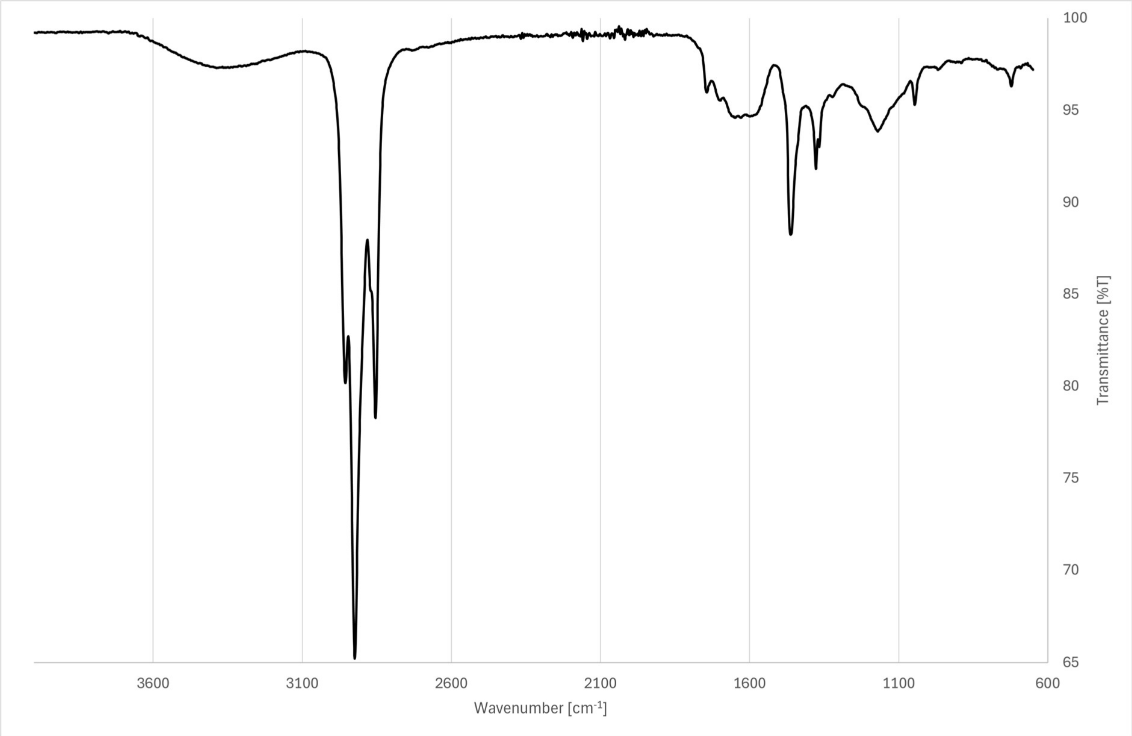

Figure S2. Full FTIR-ATR spectrum of deposits from the field injector

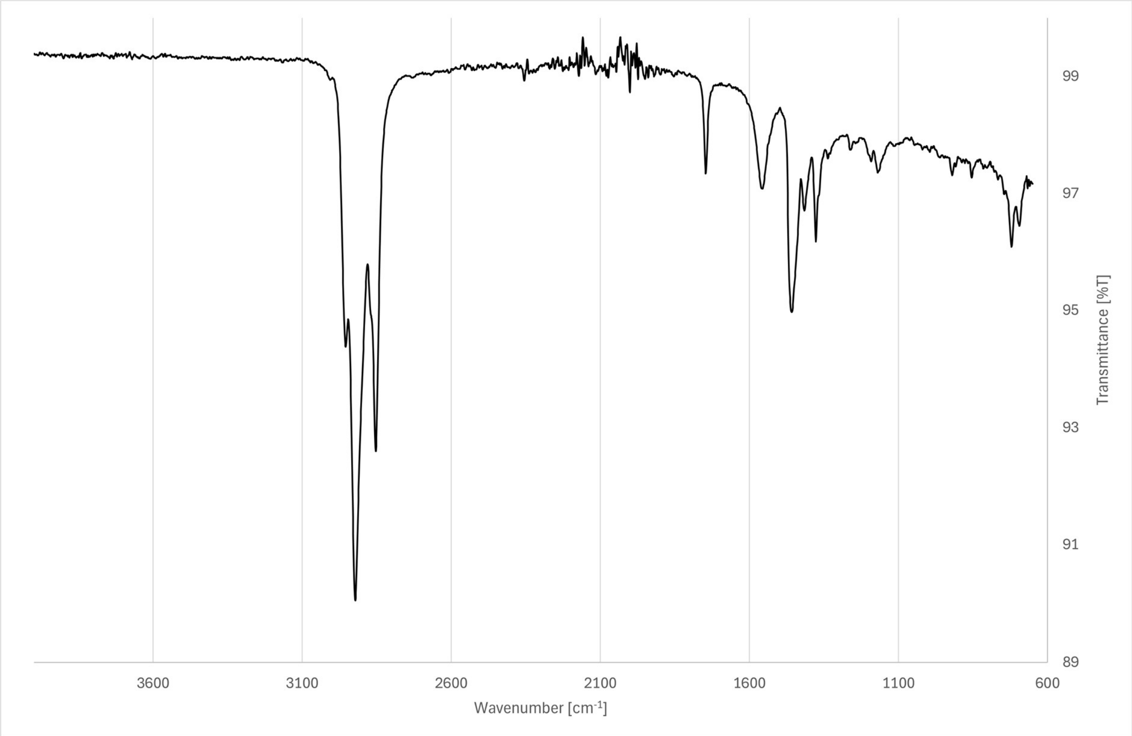

Figure S3. Full FTIR-ATR spectrum of deposits from the laboratory test rig
